# Identifying mental health discussion topic in social media community: subreddit of bipolar disorder analysis

**DOI:** 10.3389/frma.2023.1243407

**Published:** 2023-11-03

**Authors:** Tatsawan Timakum, Qing Xie, Soobin Lee

**Affiliations:** ^1^Department of Information Science, Chiang Mai Rajabhat University, Chiang Mai, Thailand; ^2^School of Management, Shenzhen Polytechnic, Shenzhen, Guangdong, China; ^3^Department of Library and Information Science, Yonsei University, Seoul, Republic of Korea

**Keywords:** bipolar, mental disorder, sentiment analysis, topic modeling, network analysis, social media data

## Abstract

Online platforms allow individuals to connect with others, share experiences, and find communities with similar interests, providing a sense of belonging and reducing feelings of isolation. Numerous previous studies examined the content of online health communities to gain insights into the sentiments surrounding mental health conditions. However, there is a noticeable gap in the research landscape, as no study has specifically concentrated on conducting an in-depth analysis or providing a comprehensive visualization of Bipolar disorder. Therefore, this study aimed to address this gap by examining the Bipolar subreddit online community, where we collected 1,460,447 posts as plain text documents for analysis. By employing LDA topic modeling and sentiment analysis, we found that the Bipolar disorder online community on Reddit discussed various aspects of the condition, including symptoms, mood swings, diagnosis, and medication. Users shared personal experiences, challenges, and coping strategies, seeking support and connection. Discussions related to therapy and medication were prevalent, emphasizing the importance of finding suitable therapists and managing medication side effects. The online community serves as a platform for seeking help, advice, and information, highlighting the role of social support in managing bipolar disorder. This study enhances our understanding of individuals living with bipolar disorder and provides valuable insights and feedback for researchers developing mental health interventions.

## Introduction

With the rapid increase in the number of social media users, social media has become a powerful tool for sharing medical information, gathering user feedback, and establishing support networks (Moorhead et al., 2013). Individuals with severe mental health problems can challenge the stigma associated with their conditions through individual empowerment and by providing hope to others through online communities. They benefit greatly from interacting with peers online, sharing personal experiences, and strategies for living with mental illness (Naslund et al., 2016). Furthermore, social media communities provide researchers with a unique opportunity to learn about patients' health experiences, treatment preferences, and potentially discover new knowledge in the field of health science. Interactions and shared information on social media offer valuable data that can shed light on the impact of drugs, diseases, and medical treatments on patients outside controlled settings.

Previous analyses compiled information on the utilization of social media for various health-related purposes, including health interventions, health promotion initiatives, medical education, and monitoring disease outbreaks (Zhao and Zhang, 2017; Marar et al., 2019; Chen and Wang, 2021). Major social media platforms like Twitter, Facebook, and Reddit have become significant sources of online health information, including mental health-related content (Zhang et al., 2013; Pershad et al., 2018; Record et al., 2018; Foufi et al., 2019). Moreover, these platforms often provide users with natural means of self-expression, including discussions about their behavior, thoughts, and emotions, which can be indicative of their emotional wellbeing (Conway and O'Connor, 2016). This abundance of user-generated content also serves as a valuable data source for researchers. Studies in the field of data mining have employed machine learning and statistical methods to analyze online messages, focusing on mood, psycholinguistic processes, and content topics extracted from posts (Nguyen et al., 2014). Patients are increasingly turning to social media platforms to search for treatments, discuss their experiences with side effects and treatments with healthcare professionals, participate in health-related online forums, and gain further knowledge about their illnesses (Monnier et al., 2002). User-generated content from social media has proven to be a rich resource for health investigations (O'Neill et al., 2014). Users' posts reflect their thoughts and feelings about their medical experiences and often attract the attention of other patients, caregivers, and medical professionals. User-generated content on the web has become a novel source of clinical data for research purposes (Denecke, 2015).

In the era of Big Data and evidence-based research, social media data offers researchers vast opportunities to gather healthcare information, including insights into patients' health behavior and medical experiences. By leveraging interdisciplinary approaches spanning social sciences, information and computer science, and applied statistics, large datasets from social media content have been integrated into biomedical and psychological research endeavors. Collaborative efforts in Big Data science can unveil and explain patterns in psychology, human behavior, cognition, and their impact on sociocultural systems over time, ultimately generating valuable data, such as medical information (Harlow and Oswald, 2016).

Previous studies utilized computer software and social media content analysis techniques to achieve various outcomes. For example, researchers collected depression and schizophrenia-related hashtags on Twitter to identify stigmatizing attitudes and personal experiences (Reavley and Pilkington, 2014), and analyzed online community posts related to mental health issues to understand the characteristics of online depression communities in terms of language styles, topics, and sentiments (Nguyen et al., 2014). Additionally, psycholinguistics was employed as a feature for text analysis to detect emotions in online communication, recognize psychological status, and predict mental conditions. For instance, a study focused on depression analysis on Facebook using machine learning and a set of psycholinguistic features to investigate the effects of depression and provide solutions for mental health problems (Islam et al., 2018). Furthermore, researchers detected posts containing words or phrases consistent with suicidal ideation on Twitter to observe the expression of suicidality based on linguistic patterns (O'Dea et al., 2015) and utilized linguistic metadata features, including specific words, parts of speech (POS), and lexicon-based elements from the Linguistic Inquiry and Word Count (LIWC), to detect depression in Reddit posts (Trotzek et al., 2018).

Similarly, social networks have provided insights into various aspects of bipolar disorder (BD), such as functioning and social skills. For example, linguistic and phonological features were applied to detect the early stages of BD from Twitter using a supervised machine-learning approach (Huang et al., 2017). Pathway analysis was employed to identify predictors of suicide ideation in older adults with BD, using socio-demographically targeted and social media advertising within Facebook and newsfeeds (O'Rourke et al., 2017). Furthermore, language impairment in Reddit was analyzed to study online mental health communities focusing on depression, BD, and schizophrenia, examining reports of positive emotion, exercising, and weight management (Park and Conway, 2018).

Numerous previous studies have examined the content of online health communities to gain insights into the sentiments surrounding mental health conditions. However, there is a noticeable gap in the research landscape, as no study has specifically concentrated on conducting an in-depth analysis or providing a comprehensive visualization of Bipolar disorder. Several researches demonstrated the association between language usage and mental disorders. Consequently, many researchers have concentrated on social media linguistics analysis and developed tools to detect language patterns that support clinical mental health care, such as systems for emotion detection from user-generated text. However, there remains a scarcity of research focusing on content and sentiment analysis.

Therefore, this study aims to explore the topics and sentiments related to mental health, specifically bipolar disorder (BD), by examining social media texts, in order to answer the following research questions:

*RQ1*. What are the main topics and discussions related to bipolar disorder within the Bipolar subreddit?
*RQ2*. What sentiments are expressed by users in the Bipolar subreddit when discussing their experiences with bipolar disorder?
*RQ3*. How does the Bipolar subreddit serve as a platform for seeking help, advice, and information related to bipolar disorder?
*RQ4*. What role does social support play in managing bipolar disorder within the Bipolar subreddit?

The findings of this study can contribute to a better understanding of individuals living with BD and offer valuable insights and feedback for researchers developing health interventions. Social media analytics can be used to track the impact and reach of health interventions in real time, allowing for the refinement and adaptation of interventions to maximize their effectiveness. Furthermore, the research framework of this study can be applied to the study of other health conditions as well.

## Materials and methods

This study aimed to examine social media discussions within Bipolar subreddit communities. The experiment utilized topic modeling and sentiment analysis to identify hidden topics and observe polarity in social media data, respectively.

### Dataset

The data used for this study was collected from the Reddit community (www.reddit.com). A finite set of two subreddits related to bipolar disorder, including https://www.reddit.com/r/bipolar and https://www.reddit.com/r/bippolar2, was selected for analysis. The subreddit “r/bipolar” community, dedicated to bipolar-related issues, has over 85.5 k members, while the subreddit “r/bipolar2” has 13.5 k members who live with bipolar disorder type 2 and encompass the entire bipolar spectrum.

**Figure 1:**
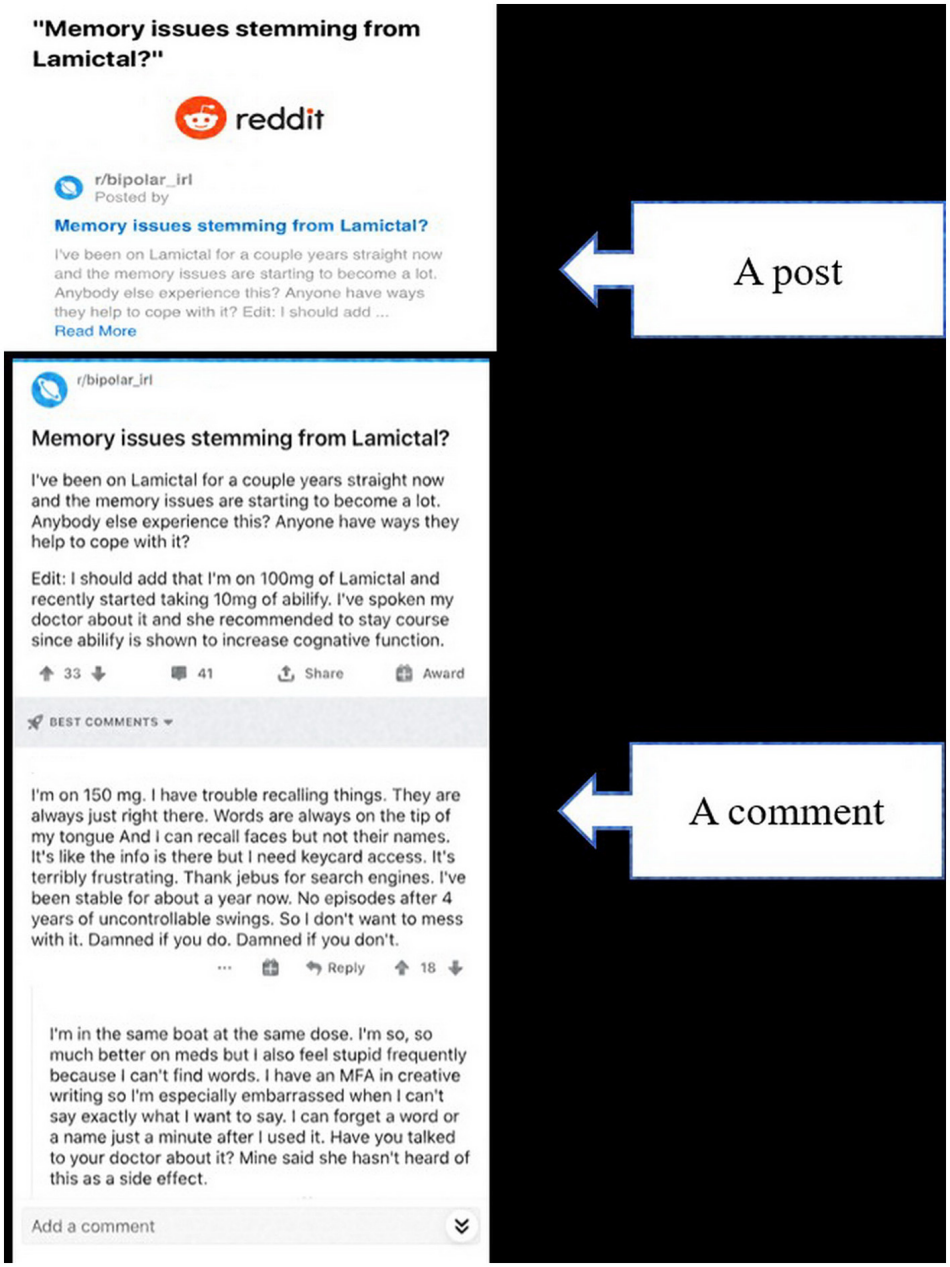
Reddit post and comment.

The posts and comments were collected from November 26th, 2018, and ended on December 28th, 2018. During this time period, a total of 1,460,447 posts were collected as plain text documents by scraping Reddit using Python Reddit API Wrapper (PRAW). Posts consist of the initial textual statements that initiate communication with other users, while comments are replies to these posts, organized in a tree-like structure (Gkotsis et al., 2016) as shown in Figure 1.

Reddit is one of the largest and most active online communities, where users can engage in discussions and share their experiences on a wide range of topics, including mental health. It boasts a large community of members, and many of them have an extensive history of previous submissions. The platform also hosts substantial content related to various diseases and medical conditions. The language of Reddit text posts is more structured than that on other major social media platforms, such as Twitter. It provides a unique environment for studying online communities due to its structure of subreddits, which are individual, topic-specific communities within the larger platform. While there may be multiple subreddits related to bipolar disorder on Reddit. We selected the “r/bipolar” and “r/bipolar2” subreddits based on factors such as their popularity, activity level, and relevance to the research objectives. Both subreddits are dedicated to in-depth discussions specifically related to bipolar disorder, making it a suitable and focused community to investigate the topics and sentiments related to this mental health condition. Furthermore, Reddit's terms and conditions allow the use of its content for research purposes, which constitutes a major advantage for researchers. The Reddit forum also tends to be distinct from similar offline groups. Users are more likely to discuss problems that they do not feel comfortable talking about in face-to-face interactions (Johnson and Ambrose, 2006).

The data used in this study was collected from Reddit, a social media platform, in accordance with the platform's terms of service and community guidelines. The data collection process strictly adhered to the ethical standards outlined by Reddit, ensuring respect for user privacy, anonymity, and the responsible use of the platform's content. Consequently, we confirm that the collection, analysis, and reuse of social media data were conducted in strict accordance with Reddit's policies and terms of use, as well as all relevant institutional regulations.

### Data processing

#### Pre-processing

Before analyzing the dataset, it underwent a pre-processing process to ensure the quality of the data prior to conducting specific analyses. This process included sentence segmentation, which involved splitting the sentences based on full-stop delimiters using the OpenNLP Sentence Detector (Apache OpenNLP Development Community, 2011). Additionally, stop word removal eliminated words that do not convey meaningful information, such as “a,” “is,” and “are.” Furthermore, Stanford CoreNLP (Manning et al., 2014) was utilized for part-of-speech tagging (POS) to identify the word types (noun, verb, adverb, and adjective) in the dataset based on tokens and context. This technique proved valuable for examining complex words and accurately determining the meaning of words within sentences. Furthermore, lemmatization was employed to analyze the lemma of each term. This step reduces inflection and derivationally related forms of words, returning them to their standard form (Manning et al., 2009; Song et al., 2015).

#### Topic analysis

The topic modeling approach was employed for content analysis to summarize the topics of bipolar disorder (BD) content in Reddit. Topic modeling is a probabilistic generative model based on the assumption that documents contain a mixture of topics consisting of the highest probability for each word. This statistical model helps identify abstract topics that occur in collections of documents. A topic represents a cluster of words that frequently appear together. In this study, we utilized Latent Dirichlet Allocation (LDA) for topic analysis, an unsupervised model that automatically clusters words into topics and associates documents with those topics. The underlying assumption is that a text document has a probability distribution over a mixture of “topics,” where each topic is associated with a distribution over words, and each word is drawn from the mixture (Blei et al., 2003).

LDA topic modeling has been a well-established and widely used technique in natural language processing and topic modeling for many years. Consequently, the availability of numerous open-source implementations, libraries, and resources for LDA makes it relatively easier to integrate into existing workflows. LDA is a relatively lightweight algorithm that can be trained efficiently on smaller datasets. LDA might be a more practical choice for tasks with limited training time and computational resources. In contrast, another approach, such as BERT Topic, being a newer approach, has fewer implementations and resources available, which could affect its adoption in certain contexts. BERT Topic is based on BERT, a large-scale language model that demands substantial computational resources and training time. Compared to BERT Topic, LDA provides a more interpretable representation of topics. LDA models assign probabilities to words in each topic, allowing users to understand and label the topics based on the most representative words. This level of interpretability is crucial in domains where human understanding and explainability are important, such as social sciences and content analysis.

**Figure 2:**
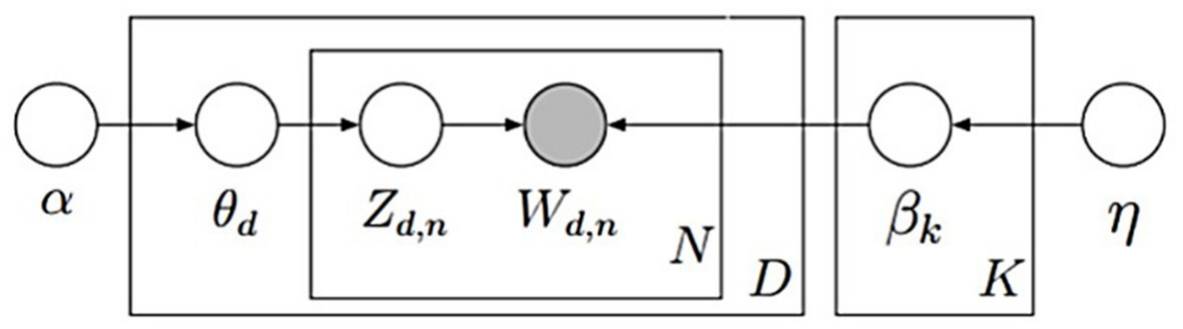
LDA topic model.

It is important to note that both LDA and the summarization algorithms assume the documents to be a “bag of words” and do not take grammar into account. This purely statistical approach relies on the meanings of documents being conveyed through words (Arora and Ravindran, 2008). Regarding the LDA topic model (shown in Figure 2), the process of generating topics for a document is as follows:

- The dirichlet distribution (α) generates the topic distribution (θ_*d*_) for document *d*.
- The multinomial distribution of the topic (θ_*d*_) assigns the topic (*z*_*d, n*_) for word *n* of document *d*.
- The corresponding topics from the dirichlet distribution (β_k_) generate the topic distribution (φ_*z*_*d, n*__).
- The multinomial distribution of topics (φ_*z*_*d, n*__) generates the words (*w*_*d, n*_).

*N* represents the different variables demonstrating the observed words in the various documents. *K* represents the total number of topics, and *D* represents the total number of documents.

Subsequently, LDA was employed for topic modeling to uncover hidden topics and terms in Reddit posts. This process analyzes the extensive amount of social media text to determine the connections between topics and summarizes the dataset on a scale that is impossible to achieve through manual annotation. In this study, we configured the algorithm to identify 30 topics per dataset, with each topic displaying its top 30 words.

The LDA algorithm was employed to perform clustering, aiming to identify coherent topics within the dataset. It was observed that a higher coherence score yielded better clustering results. Coherence served as a metric for determining the optimal number of clusters. The coherence scores were calculated using the scikit-learn metric package. We conducted experiments by varying the number of cluster parameters from 5 to 50, with intervals of 5. Among these values, the coherence score was highest when the number of topics (K) was set to 10. However, we conducted a manual inspection of the data. Upon manual inspection, it became evident that the results were most distinct and interpretable when the *K*-value was set to 30. Smaller *K*-values, such as 5 and 10, rendered the characteristics of the topics unclear and incomplete. Conversely, the largest *K*-value (50) resulted in an overlap of topic words across most topics. Consequently, we determined the optimal number of topics to be 30 for the overview topics and to be 20 for the sentiment topics.

Finally, we considered the list of top words in the probability distribution for each topic to assign a name to that specific topic. In other words, this interpretative process of labeling LDA topic outputs involves selecting a set of words from each topic's probability distribution that best represents the theme or subject of that topic. These words essentially serve as labels or names for the topics. Therefore, this process entails selecting words from each topic's probability distribution to provide a meaningful name or label that captures its content or theme. Afterward, we measured the agreement among three annotators regarding the labeling of the topics.

#### Sentiment analysis

An essential task in sentiment analysis is to classify the polarity of a given text at the sentence or document level, determining whether the expressed opinion is positive, negative, or neutral. Advanced sentiment classification can also identify emotions such as happiness, sadness, and anger, providing insights into the psychological patterns in text based on the analysis of human verbal behavior (Gottschalk and Bechtel, 2008). Therefore, sentiment analysis examines sentiment and detects individual words and phrases in the text to reflect the emotional scales.

To investigate the polarity properties of social media posts from Reddit, the SentiStrength software (http://sentistrength.wlv.ac.uk/download.html) was utilized for automatic sentiment analysis. SentiStrength is specifically designed for analyzing social web texts and predicts the strength of positive and negative sentiment in short texts, even for informal language, by employing a lexical approach. The sentiment analysis captures reactions to events through free texts, offering new insights into human behavior and people's positive or negative attitudes toward an event (Thelwall and Buckley, 2013). The software operates in two modes: supervised and unsupervised. In the unsupervised mode, it employs pre-defined sentiment strength weights for the lexicon, while in the supervised mode, it utilizes a training set of data to automatically adjust the lexicon term weights and produce more accurate results.

At the core of SentiStrength is a list of 2,608 words and word stems, each associated with a typical positive or negative sentiment strength for social web texts. The software assigns a positive sentiment strength ranging from 1 (no positive sentiment) to 5 (very strong positive sentiment) and a negative sentiment strength ranging from −1 (no negative sentiment) to −5 (very strong negative sentiment) to each text (zeros are not used). In the presence of multiple sentiment words, the strongest sentiment word is chosen, while in the absence of sentiment words, a neutral sentiment is assumed. Additionally, the software incorporates special rules for handling negations, questions, booster words (e.g., “very”), emoticons, and various other special cases (Thelwall et al., 2010, 2012).

## Results

### Bipolar disorder topic modeling

To analyze the content of Reddit data, we assume that the number of 30 topics in LDA corresponds to the number of issues or events that describe each dataset. Therefore, we fixed *K* = 30 throughout this study. For each topic, we present only 30 words with higher probabilities under that topic.

In this paper, we display the top 10 ranked topics based on the topic probability distribution scores. Each topic consists of 30 words, with stronger influences indicating higher relevance to the topic. Using this information, we manually labeled each topic with a corresponding word. Additionally, we removed groups of words that appeared as stand-alone words or special characters, as they did not provide any relevant information about the topic. We replaced them with the next word in the rank. The main idea of each dataset's content is provided below.

The top 10 topics related to bipolar disorder (BD) and their top 30 words are as follows:

Topic 1. Help and advice (0.161): Social media users shared their conditions, asked for help and information, and expressed appreciation.

Topic 2. Bipolar disorder (0.158): Discussions about symptoms, mood swings, diagnosis, and medication.

Topic 3. Social process (0.139): Patients expressed the need for mental support from their social environment, such as family and friends.

Topic 4. Therapy (0.120): Experiences shared regarding medicine, doctors, and psychiatrists.

Topic 5. Drugs (0.116): Discussions about medications used for BD and related mental health issues on Reddit, including Lithium, Lamictal, and Wellbutrin.

Topic 6. Episode (0.084): Personal accounts of specific periods or conditions experienced by individuals living with BD.

Topic 7. Emotion expression (0.059): Patients with BD expressed their feelings about living with the illness, including emotions such as hate, sadness, and crying.

Topic 8. Friend (0.054): Frequent mentions of individuals who interacted with patients in social settings.

Topic 9. Relaxation (0.047): Techniques shared for relaxation during times of stress and anxiety, such as smoking, drinking, taking drugs, sleeping, eating, and meditation.

Topic 10. Drug-side effect (0.045): Discussions about the side effects of drugs used for BD, with weight gain being the most frequently mentioned side effect (Table 1).

**Table 1:** Top-10 topics of bipolar disorder discussion in the Reddit communities.

**Topic label**	**Topic 1**		**Topic 2**		**Topic 3**		**Topic 4**		**Topic 5**	
	**Help and advice**		**Bipolar disorder**		**Social process**		**Therapy**		**Drugs**	
Probability scores	0.16115		0.1582		0.13998		0.12064		0.1164	
Frequency of words	Thank Thanks Hear Glad Help I-hope Sorry FEEL Share Post Try I-feel Look Hope Reply Definitely Hard Comment Read Nice Happy Time Sound Advice Luck Word Start Helpful Sub Appreciate	63,864 30,261 22,372 22,207 20,575 17,930 16,814 15,686 14,531 12,577 11,852 11,619 11,398 10,149 8,317 8,170 7,448 7,353 7,349 7,274 7,233 7,107 7,066 6,974 6,405 5,795 5,719 5,633 5,507 5,480	Bipolar Depression Mood Diagnose Hypomanic Mixed Hypomania Feel Manic Experience Diagnosis Depressive Mania Time Mixed-episode Doctor Psychiatrist Symptom Episode Medication I-feel Depressive-episode Start Bipolar-disorder Manic-episode Anxiety Rapid Rapid-cycling Happen Med	82,296 38,808 33,029 32,433 23,530 21,250 20,783 20,517 20,183 18,888 17,897 17,731 17,601 17,131 16,853 15,534 15,424 15,310 15,059 14,658 14,625 13,299 10,787 10,571 10,052 9,670 9,606 9,491 9,208 8,363	Bipolar Help Feel Time Tell Friend Love Life Try Relationship yourself Support Understand Hard Mental Talk Care Person Family I-feel Mean Deal I-hope Learn Diagnosis Sound Illness Feeling Hurt Mental-illness	33,047 29,800 29,300 25,765 24,328 24,168 22,309 21,434 20,360 19,564 19,170 19,095 18,129 16,953 15,527 15,490 14,930 14,107 13,691 12,972 12,851 10,732 10,461 9,668 8,958 8,542 8,348 8,027 7,983 7,829	Med Help Doctor Try Feel Time Medication Psychiatrist Life Bipolar Therapist Ago Luck Talk Tell Start Diagnosis Help-i Symptom Mood Pdoc Stop Diagnose Doc Effect Depression Prescribe Pill Appointment Therapy	74,865 30,637 23,993 22,148 19,591 19,086 17,821 15,681 14,047 10,744 9,408 8,997 8,426 8,400 8,054 7,940 7,911 7,240 7,113 6,975 6,642 6,599 6,233 6,188 5,950 5,486 5,360 5,348 5,323 4,927	Lithium Lamictal Effect Dose Doctor Med Medication Start Mood Try Help Time Wellbutrin Doc Experience Dosage Feel Psychiatrist Low Stop Prescribe Lamotrigine Pdoc Depression Increase Mood-stabilizer Notice Anxiety Add Drug	42,276 40,493 39,410 24,659 23,847 19,571 19,000 17,773 16,725 14,625 14,529 13,106 12,825 12,065 11,876 11,590 10,685 10,085 9,934 9,426 9,250 9,159 8,941 8,885 8,304 7,867 7,810 7,704 7,563 7,260
Probability scores	0.08456		0.05908		0.05406		0.04717		0.04573	
Frequency of words	Sleep Night Bed Morning Time Feel I-feel Hour Help Wake I-start I-wake Fall Try Seroquel I-sleep Happen Else Fall-asleep Energy Start Tired Stay Little Super Couple Dream Tell Med Body	59,445 38,903 17,855 15,444 15,071 14,059 12,268 11,709 10,571 10,461 8,395 7,428 6,829 6,786 6,740 6,511 6,472 5,945 5,737 5,643 5,523 5,261 5,145 5,027 4,623 4,420 4,138 4,094 3,978 3,850	Feel I-feel Time I-hate Try Life Wanna Fuck Shit Bad Wan Tell Deal Happen Med Help Die Cry Stop Sometimes Kill Stay Friend Brain Totally Feeling Care Else Live Depression	17,039 16,756 12,838 10,471 9,214 7,246 6,948 6,236 5,854 5,510 5,509 4,627 4,527 4,378 4,257 3,955 3,864 3,573 3,489 3,437 3,428 3,376 3,278 3,247 3,175 3,151 2,984 2,938 2,838 2,811	I-feel Feel Time Friend Help Life Feeling Love Tell Try Happen Person Little Talk Bring Sometimes-i Hard Bad Voice Idea Else Maybe Leave Help-i College Look Start Head Depression Word	16,101 14,878 12,789 7,550 6,715 5,837 5,134 4,977 4,445 4,103 3,814 3,697 3,692 3,632 3,627 3,598 3,417 3,183 3,088 3,064 3,058 3,054 2,925 2,822 2,763 2,760 2,730 2,612 2,525 2,508	Help Smoke Time Anxiety Weed Try Help-i Drink I-feel Feel Life Stop Alcohol Panic Start Sleep Drug Medication Med I-smoke Eat Bit Smoking hard Panic-attack Job Bad Worse Manic Quit	16,821 12,094 11,977 11,081 10,436 7,876 7,398 7,210 6,880 6,769 5,635 4,985 4,739 4,729 4,724 4,453 4,432 3,895 3,840 3,806 3,805 3,740 3,693 3,509 3,230 3,207 3,133 3,099 3,013 2,904	Weight Abilify Weight-gain Eat Med Gain Lamictal Latuda Effect Lose Time Start Seroquel Gain_weight I-gain Help Body Stop Feel Food I-feel Depression Try Switch Appetite Medication I-eat Cause Pretty Little	17,854 12,770 12,089 11,338 10,922 10,757 9,343 9,266 8,781 7,043 6,588 5,866 5,708 5,687 5,435 4,586 4,223 4,122 3,732 3,570 3,501 3,485 3,406 3,302 3,153 3,152 3,130 3,059 2,969 2,692

### Bipolar disorder conversation sentiment analysis

By employing topic-based sentiment analysis on social media data, it becomes possible to identify people's moods, related issues, and emotional responses to specific situations. For instance, when discussing a health topic, the mention of a drug name often evokes strong positive or negative sentiments within the conversation. To gauge the emotional intensity within the bipolar disorder (BD) community, we utilized SentiStrength 2.3 for sentiment analysis. This tool adopts a lexical approach that begins with a collection of terms known to have sentiment associations. It then applies a set of rules to predict the sentiment of texts based on the occurrence of these words. After obtaining the results of positive and negative text classification, we integrated these findings into the topic modeling approach to determine the common topics associated with each polarity.

### Polarity classification results

The system employed sentiment analysis to determine the sentiment strength in Reddit data, categorizing each text as neutral, positive, or negative. However, for the purposes of this study, we focused solely on reporting the positive and negative sentiment text to observe the experiences related to bipolar disorder. Each text was assigned two numerical values by SentiStrength: a score between 1 and 5 for positive sentiment strength, and a score between −1 and −5 for negative sentiment strength. Additionally, a score of 1 or −1 indicated no sentiment, while a score of 5 or −5 represented a strong sentiment of that type. For example, a social web text with a score of 1–4 would convey weak positive sentiment and strong negative sentiment. The system utilized two scales because a conviction can encompass both positivity and negativity, and the objective was to detect the expressed viewpoint rather than the overall polarity (Thelwall et al., 2010). Below are some examples of the short text analysis results:

**“*Cried at work Cried at work”***
***(Negative)* 1** **−4** Cried [−3] at [0] work [0] [[Sentence=-4,1=word max,1–5]] [[[1, −4 max of sentences]]]
**“*Wow, fantastic response. Thank you so much for sharing, you encouraged*** ***me!”***
***(Positive)* 4** –**1** Wow[2] fantastic[2][+1 Multiple Positive Words] response[0] [[Sentence=-1,4=word max, 1–5]] Thank [1] you [0] so [0] much [0] For [0] sharing [0] you [0] encouraged [1] me [0] [[Sentence= −1,2=word max, 1–5]] [[[4, −1 max of sentences]]]

The software assigned a score to each word in the sentence, and a total weighted score was calculated to predict the text's polarity. Each word received a numerical value based on its strength. For instance, the word “cried” was assigned a strongly negative score of −3, while both “wow” and “fantastic” were rated as moderately positive with a score of 2.

The results of the sentiment analysis indicate that there are more negative sentiment (641,264) than positive sentiment (392,352) within the discussions of Reddit-related bipolar disorder communities. Examples of the experimental results are presented in Table 2.

**Table 2:** Examples of polarity categorization.

**Reddit text post**	**Positive score**	**Negative score**	**Polarity**	**Weighted scores**
Glad it helped somehow somehow Even if originally bio-chemical, the “real” effects of bipolar affect us in deep psychological ways too.	2	−1	Positive	Glad[1] it[0] helped[0] somehow[0][+1 Emoticon] Even[0] if[0] originally[0] bio[0] chemical[0] the[0] real[0] effects[0] of[0] bipolar[0] affect[0] us[0] in[0] deep[0] psychological[0] ways[0] too[0] [[Sentence=-1,2=word max, 1–5]][[[2,−1 max of sentences]]]
Do you have a therapist you work with? Can you get an appointment soon? 10 h days consistently is a lot for anyone, maybe give yourself a break from that many hours. You need to be your best friend and advocate even though that is one of the hardest things to do. Do you have a friend or family member you can talk to? Sending you hugs.	3	−1	Positive	Do[0] you[0] have[0] a[0] therapist[0] you[0] work[0] with[0] [[Sentence=-1,1=word max, 1–5]] Can[0] you[0] get[0] an[0] appointment[0] soon[0] [[Sentence=-1,1=word max, 1–5]] 10hr[0] days[0] consistently[0] is[0] a[0] lot[0] for[0] anyone[0] maybe[0] give[0] yourself[0] a[0] break[0] from[0] that[0] many[0] hours[0] [[Sentence=-1,1=word max, 1–5]] You[0] need[0] to[0] be[0] your[0] best[1] friend[1] and[0] advocate[0] even[0] though[0] that[0] is[0] one[0] of[0] the[0] hardest[0] things[0] to[0] do[0] [[Sentence=-1,2=word max, 1–5]] Do[0] you[0] have[0] a[0] friend[1] or[0] family[0] member[0] you[0] can[0] talk[0] to[0] [[Sentence=-1,2=word max, 1–5]] Sending[0] you[0] hugs[2] [[Sentence=-1,3=word max, 1–5]][[[3,−1 max of sentences]]]
I actually just purchased a Bipolar II specific workbook that goes through CBT, DBT, and mindfulness, I'm really excited to get it. I'm glad they've helped you!	4	−1	Positive	I[0] actually[0] just[0] purchased[0] a[0] Bipolar[0] II/I[0] specific[0] workbook[0] that[0] goes[0] through[0] CBT[0] DBT[0] and[0] mindfulness[0] I'm[0] really[0] excited[2][1 LastWordBoosterStrength] to[0] get[0] it[0] [[Sentence=-1,4=word max, 1–5]] I'm[0] glad[1] they've[0] helped[0] you[0] [[Sentence=-1,2=word max, 1–5]][[[4,−1 max of sentences]]]
That's adorable!! Also try sudoku, that's supposed to be a good memory builder. My therapist also says to start journaling during the day because writing things down can leave a thicker mark on the memory. Good luck :)	5	−1	Positive	That's[0] adorable[3][+0.6 EmphasisInPunctuation] [[Sentence=-1,5=word max, 1–5]] Also[0] try[0] sudoku[0] that's[0] supposed[0] to[0] be[0] a[0] good[1] memory[0] builder[0] [[Sentence=-1,2=word max, 1–5]] My[0] therapist[0] also[0] says[0] to[0] start[0] journaling[0] during[0] the[0] day[0] because[0] writing[0] things[0] down[0] can[0] leave[0] a[0] thicker[0] mark[0] on[0] the[0] memory[0] [[Sentence=-1,1=word max, 1–5]] Good[1] luck[2][+1 Emoticon] [[Sentence=-1,4=word max, 1–5]][[[5,−1 max of sentences]]]
Wondering about life w/o meds	1	−1	Neutral	Wondering[0] about[0] life[0] w/o[0] meds[0] [[Sentence=-1,1=word max, 1–5]][[[1,−1 max of sentences]]]
I forgot to add, that the lower the dosage of the Seroquel, the drowsier you are. My effective dose was 300 mg, but I was still so tired. We bumped me up to 400 mg so I could sleep 10 h instead of 12. In addition, make sure you're taking the pill 3–4 h prior to bedtime so you concentrate the drowsiness (as much as possible) during your sleeping hours. I take mine at 5 p.m. for this reason. Just keep backing it off until the timing is right for you.	1	−2	Negative	I[0] forgot[0] to[0] add[0] that[0] the[0] lower[0] the[0] dosage[0] of[0] the[0] Seroquel[0] the[0] drowsier[0] you[0] are[0] [[Sentence=-1,1=word max, 1–5]] My[0] effective[0] dose[0] was[0] 300 mg[0] but[0] I[0] was[0] still[0] so[0] tired[−1] [[Sentence=-2,1=word max, 1–5]] We[0] bumped[0] me[0] up[0] to[0] 400 mg[0] so[0] I[0] could[0] sleep[0] 10[0] hours[0] instead[0] of[0] 12[0] [[Sentence=-1,1=word max, 1–5]] And[0] make[0] sure[0] you're[0] taking[0] the[0] pill[0] 3[0] −4[0] hour[0] prior[0] to[0] bedtime[0] so[0] you[0] concentrate[0] the[0] drowsiness[−1] as[0] much[0] as[0] possible[0] during[0] your[0] sleeping[0] hours[0] [[Sentence=-2,1=word max, 1–5]] I[0] take[0] mine[0] at[0] 5pm[0] for[0] this[0] reason[0] [[Sentence=-1,1=word max, 1–5]] Just[0] keep[0] backing[0] it[0] off[0] until[0] the[0] timing[0] is[0] right[0] for[0] you[0] [[Sentence=-1,1=word max, 1–5]][[[1,−2 max of sentences]]]
Lack of appetite and weight loss with Lamictal	1	−3	Negative	Lack[−1] of[0] appetite[0] and[0] weight[0] loss[−2] with[0] Lamictal[0] [[Sentence=-3,1=word max, 1–5]][[[1,−3 max of sentences]]]
Does anyone else feel the difference between suicidal depressed and regular depressed	1	−4	Negative	Does[0] anyone[0] else[0] feel[0] the[0] difference[0] between[0] suicidal[0] depressed[−3] and[0] regular[0] depressed[−3] [[Sentence=-4,1=word max, 1–5]][[[1,−4 max of sentences]]]
For those of you out there feeling sad or depressed, hopeless or helpless, anxious or suicidal, self-harming or self-medicating, or suffering from mental illness	1	−5	Negative	For[0] those[0] of[0] you[0] out[0] there[0] feeling[0] sad[−3] or[0] depressed[−3] hopeless[−3][−1 MultiplePositiveWords] or[0] helpless[−1] anxious[−2] or[0] suicidal[0] self[0] harming[−2] or[0] self[0] medicating[0] or[0] suffering[−3] from[0] mental[0] illness[−1] [[Sentence=-5,1=word max, 1–5]][[[1,−5 max of sentences]]]

### Polarity topics results

In order to investigate the polarity of emotions surrounding BD in social media discourse, we identified common issues that were publicly discussed within the Reddit-related bipolar community. The texts from the sentiment analysis results were divided into two datasets: positive and negative. These datasets were then subjected to LDA topic modeling to uncover hidden topics within each polarity. We set the number of topics to 20 and specified that each topic should consist of 20 words with the highest probabilities.

The LDA results present the content of documents with 20 different topic mixtures and 20 words per topic. For the purpose of this paper, we selected the top five topics to report. We manually assigned labels to these top five topics based on the words associated with each topic.

Table 3 illustrates the positively-related topics of BD within the Reddit community. These topics include “Medication”, where users express their feelings and opinions regarding drugs used in bipolar treatment, such as lamictal, abilify, and lithium, and discuss their effectiveness. The next topic is “Social wellbeing”, which explores the sense of social inclusion, individuals' lifestyles, and overall quality of life. The third topic is “Personal concerns”, which delves into social factors with a greater focus on personal experiences, such as career, relationships, and children. The fourth topic is “Social support”, which highlights the importance of having friends, family, and others to rely on during times of need and who provide encouragement when experiencing bipolar-related symptoms, such as mood swings. Lastly, the fifth topic is “Mental therapy”, which revolves around discussions within the community regarding various treatments for the disease.

**Table 3:** Top-five positive topics.

**Topics**	**Top words**
Medication (0.00278)	Lamictal weight side medication abilify seroquel lithium latuda wellbutrin luck gain doctor dose night mood hope food body sleep doc
Social wellbeing (0.00266)	Awesome life happy glad advice feeling social word sense hard love step healthy high hope issue night long friend heart
Personal concerns (0.00194)	List idea hug daylio emood app kid mood love work free vibes life easy feeling relationship luck mind minute
Social support (0.00189)	Episode glad mixed real mania parent life friend manic quick psychiatrist hypomanic luck person family idea normal partner support story
Mental therapy (0.00187)	Therapy life therapist mental helpful disorder person health idea interesting luck diagnosis hard job question feeling dbt super problem group

Table 4 presents the negative topics of BD in the Reddit community. The first topic is “Drugs”, where social media users share their experiences related to medications and their side effects. The following topic is “Mood swings”, where individuals living with bipolar disorder describe their experiences with mixed episodes, including depression, hypomania, and mania. The next topic is “Bipolar symptoms”, which encompasses the various symptoms associated with the condition, such as anxiety, depression, panic, and happiness, typically occurring during the night and morning and significantly impacting their lives. The fourth topic is “Treatments”, where individuals discuss their attempts to address negative feelings through strategies like meeting friends, consulting with a psychiatrist, and seeking medical assistance. Lastly, the fifth topic discussed within the bipolar sub-Reddit community is “Mental disorder”, which takes a broader perspective on the mental challenges they face in both personal and social aspects of their lives.

**Table 4:** Top-five negative topics.

**Topics**	**Top words**
Drugs (0.00915)	Lamictal dose side depression medication anxiety lithium doctor weight abilify latuda wellbutrin mood seroquel psychiatrist night gain low drug stabilizer
Mood swings (0.00912)	Episode depression mood symptom mania manic depressive hypomanic hypomania diagnosis mixed anxiety psychiatrist medication disorder depressed normal experience
Symptoms (0.00428)	Night sleep anxiety depression shit work feeling friend tired episode normal body morning panic attack bed happy brain full life
Treatments (0.00409)	Life thought night feeling friend bed medication episode depression anxiety therapist depressed work doctor psychiatrist morning hard manic therapy dose
Mental disorder (0.00369)	Disorder therapist mental diagnosis therapy mood life depression anxiety medication issue illness psychiatrist doctor symptom bpd reason health episode relationship

**Figure 3:**
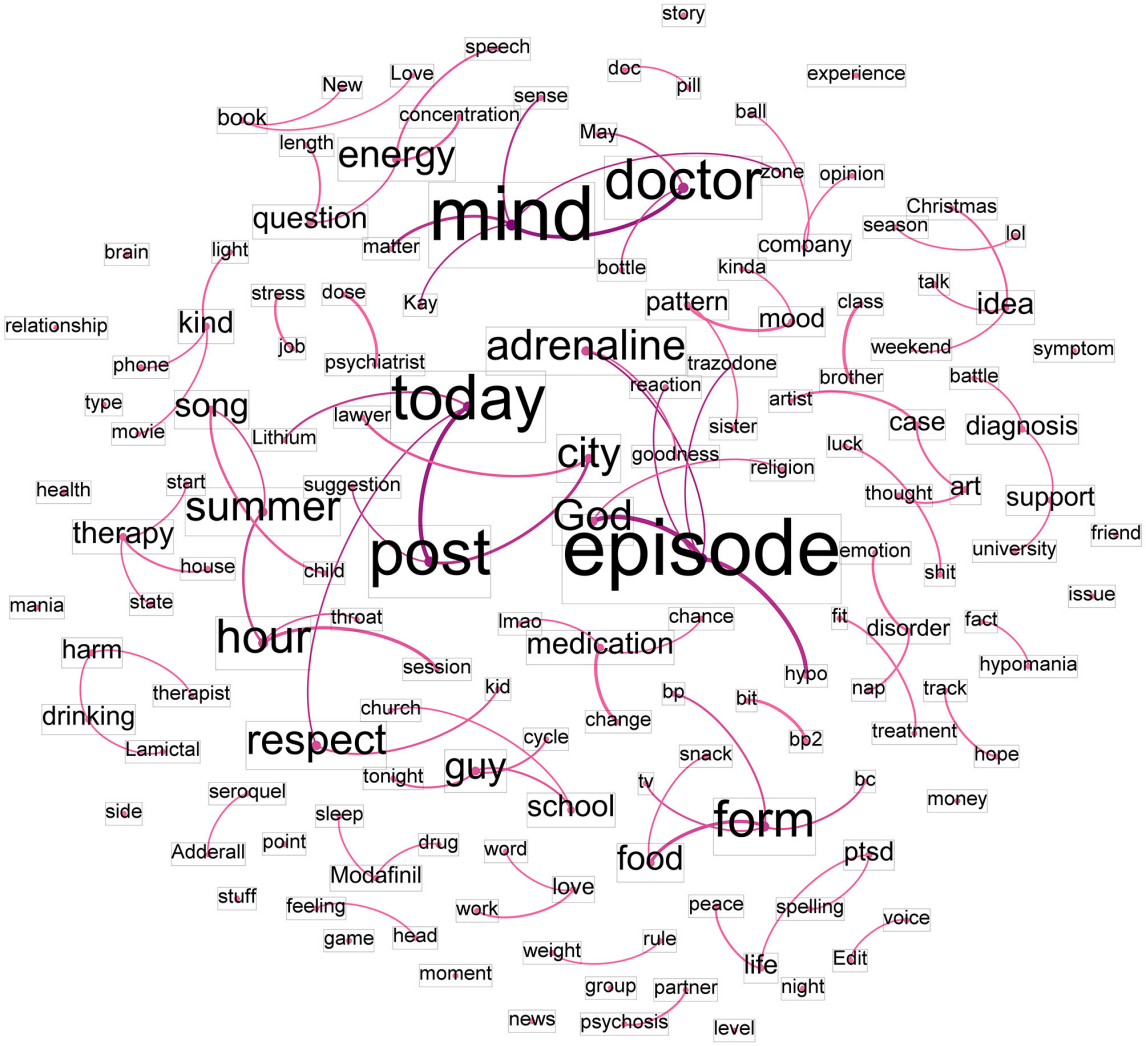
Network of co-occurring words in the positive post text dataset.

To illustrate the association between terms and document topics, we constructed a co-word map. The positive and negative text post were processed into graphML format to generate word co-occurrences and create a network of co-occurring words. Subsequently, we visualized the graph using Gephi software, adjusting the nodes to highlight those with high betweenness centrality for improved visualization. This allowed us to observe the graphs with key terms (nodes) representing the core concepts within each polarity. The results are presented below:

Figure 3 presents the co-word network of the positive post text. The terms that influenced other nodes the most were “life” (0.00016), followed by “mind” (0.000149), “doctor” (0.000142), “episode” (0.000118), and “medication” (0.000114). These terms exhibited a high betweenness centrality score. The co-word network confirms a core concept of positive conversations, where Reddit users shared experiences about their personal lives, including medication, mind therapy, activities, and social environments. Additionally, the network reveals connected terms such as life-peace, doctor-mind, work-love, medication-change, and song-summer.

**Figure 4:**
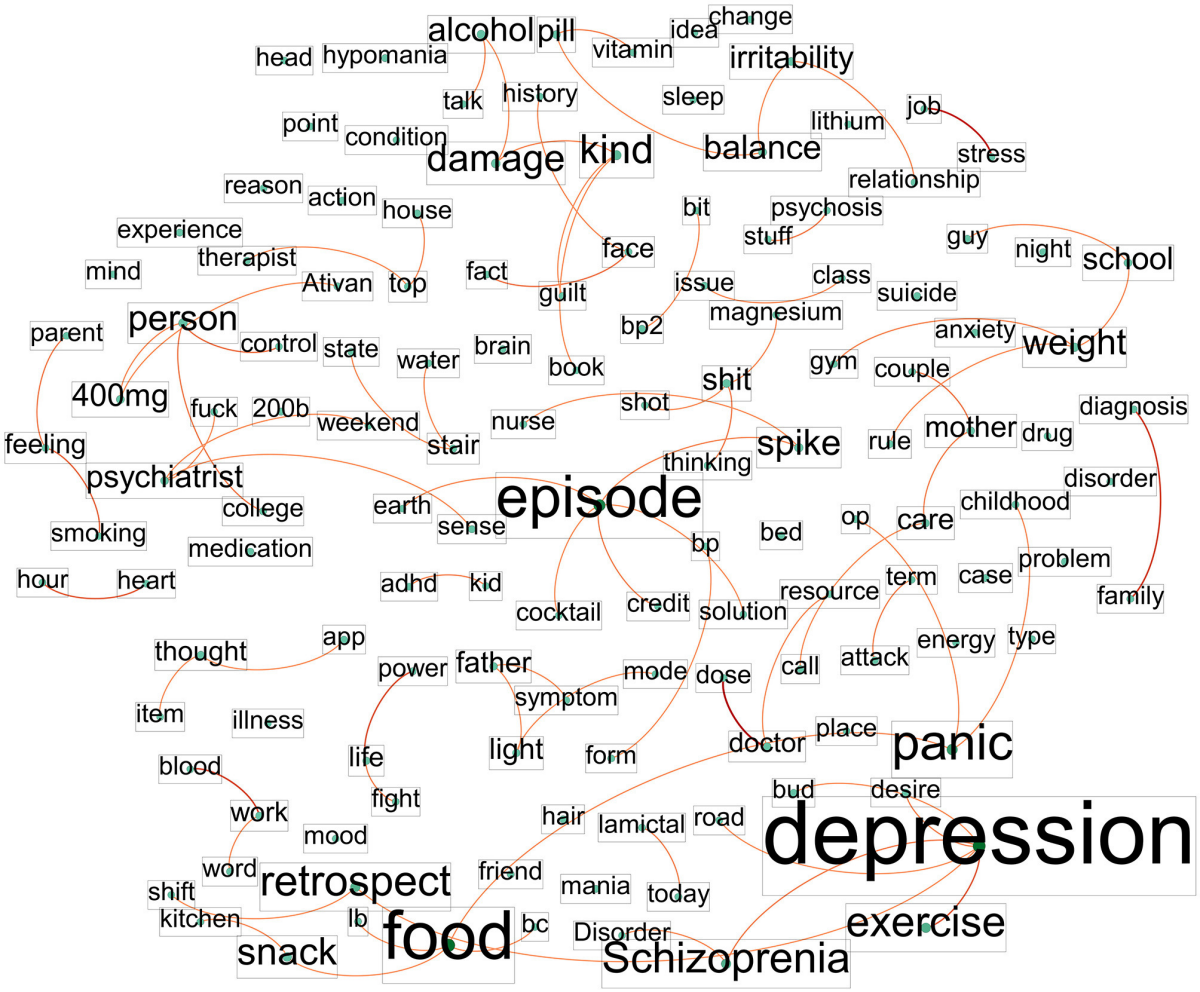
Network of co-occurring words in the negative post text dataset.

Figure 4 displays the co-occurrence network of words in the negative post text. The top-ranking terms, which play a crucial role as bridges in the network, are “depression” (0.000256), followed by “life” (0.000164), “episode” (0.000157), “shit” (0.000101), “psychiatrist” (0.000093), and “food” (0.000091). This graph reveals that individuals living with BD expressed concerns about recurrent disorders such as schizophrenia and obesity, along with symptoms of mood swings. Furthermore, they experienced irritability in their relationships with others and struggled with negative thoughts, including suicidal ideation. Notable word pairs identified in the network include depression-schizophrenia, life-fight, episode-spike, shit-thinking, food-panic, and irritability-relationship.

## Discussion

### Topics discussed in the bipolar disorder online community of Reddit users

The online community of Reddit users discussing Bipolar disorder covers a variety of topics related to the condition. The top 10 topics predominantly revolve around symptoms, mood swings, diagnosis, and medication. Users openly share their personal experiences with bipolar episodes, including the challenges they face and strategies for coping. They also express their emotions about living with bipolar disorder, seeking solace and connection with others who can relate to their struggles. Therapy is another crucial topic, with users sharing their experiences and interactions with mental health professionals. They discuss different therapeutic approaches, the benefits of therapy in managing bipolar disorder, and provide tips for finding a suitable therapist.

Discussions related to medication are common, as users share their experiences with specific medications such as Lithium, Lamictal, and Wellbutrin. They talk about side effects and how certain drugs have helped them manage their condition. Weight gain is often mentioned as a common side effect, and users seek advice and strategies for managing medication-related effects.

The findings demonstrate that a large number of social media users use online community platforms to discuss mental health issues. Users have a keen interest in seeking out information related to treatments, engaging in conversations with others to express their thoughts on the efficacy and potential side effects of treatments, participating in discussions within mental health communities to address their queries, and acquiring insights about their medical conditions (Nguyen et al., 2014; Reavley and Pilkington, 2014; Foufi et al., 2019).

Social support plays a significant role in the management of bipolar disorder. Within the online community, users engage in discussions about effective communication with loved ones regarding the condition, seeking understanding, empathy, and establishing a support system. Friends are regarded as essential in the lives of individuals with bipolar disorder, and conversations often revolve around the impact of friendships on mental health, maintaining relationships, and the importance of supportive friends. This finding is consistent with a study on the use of social media for social support among adolescents, including emotional, appraisal, and informational support (Selkie et al., 2020). Discussions also cover various relaxation techniques aimed at managing stress and anxiety associated with bipolar disorder. Methods such as smoking, drinking, medication use, sleeping, eating, and meditation practices are mentioned. It is important to approach coping strategies, including substance use, with caution and consult healthcare professionals for guidance. Moreover, the online community serves as a platform for seeking help and information. Users actively seek advice on managing symptoms, finding effective treatment options, and expressing gratitude for the support they receive. Social media enables people to share information on treatments and research to improve care (De Choudhury and De, 2014; Pershad et al., 2018).

Overall, the Bipolar disorder online community on Reddit encompasses a wide range of topics related to the condition, fostering support, understanding, and the sharing of experiences among its members.

### Polarity classification

In the analysis of people's opinions and feelings toward mental health issues, specifically related to Bipolar Disorder (BD), the sentiment was assessed using the SentiStrength software. The software analyzed user-generated text from social media, which described situations related to health problems. The results of the sentiment analysis on the Reddit dataset, under the topic of BD indicated a higher frequency of negative polarity compared to positive polarity. This finding suggests that discussions and expressions within the Bipolar Disorder online community on Reddit tended to lean toward a more negative sentiment. It indicates that individuals sharing their experiences, challenges, and emotions related to BD often conveyed negative feelings or perspectives.

It is important to note that sentiment analysis provides an automated assessment of sentiment based on textual content, and it may not fully capture the nuances and complexities of individual experiences. Additionally, the analysis is limited to the specific dataset and platform analyzed, and sentiments expressed may vary across different social media platforms and online communities. For instance, using Twitter content analysis to measure attitudes toward mental illness and analyzing mental health based on the positive language of Facebook users provided different insights into online discussions about mental health (Reavley and Pilkington, 2014; Bogolyubova et al., 2020).

Further qualitative analysis and a deeper understanding of the specific context and content of the negative sentiment would be necessary to gain insights into the concerns, challenges, and negative experiences expressed by individuals living with or relating to Bipolar Disorder in the Reddit community.

### Polarity topics

The study findings revealed that individuals expressed their thoughts and emotions related to both the biological and mental aspects of their conditions. Distinct topics emerged when analyzing positive and negative sentiments. Regarding positive sentiment, Reddit users discussed receiving encouragement and support from various sources, including friends, partners, family members, doctors, and psychiatrists. They expressed satisfaction with their social wellbeing while living with a mental illness. Some users also reported positive experiences with bipolar medication and psychological therapy, although others mentioned negative effects such as weight gain from the drugs.

On the other hand, negative sentiment topics focused on the negative aspects of drugs and treatments, including instances of incorrect diagnosis by physicians when experiencing mental symptoms. Users also shared their struggles with bipolar disorder symptoms, particularly mood swings, which caused both biological, and psychological stress.

The polarity findings emphasize the results showing that social media users seek information to address their mental health problems and to connect with individuals who can understand and encourage positive emotions. These findings underscore the importance of addressing patients' wellbeing and providing appropriate treatments for their mental health.

## Limitation

It is essential to acknowledge certain limitations inherent in our study. One notable limitation is the nature of bipolar disorder (BD) as a clinical diagnosis. Within the context of our research, participants on social media platforms may encompass a diverse range of individuals, including those who have received a professional diagnosis of BD, those who self-identify as bipolar, and individuals who are family members of those with BD. Consequently, this gap introduces a potential risk of misattribution when analyzing sentiments and topics related to BD within the context of discussions about BD. It is important to recognize and consider this limitation.

Furthermore, in future work, we should consider the potential value of predicting user post-behavior during the presence of mental symptoms and conducting a sentiment comparison between Bipolar Disorder (BD) and other online communities.

## Conclusion

This study aimed to explore the topics and sentiments discussed in the Bipolar disorder online community on Reddit. The findings revealed that users engaged in discussions related to various aspects of bipolar disorder, including symptoms, mood swings, diagnosis, medication, therapy, social support, and relaxation techniques. They shared their personal experiences, sought advice, and expressed their emotions about living with bipolar disorder. The analysis showed a predominance of negative sentiment expressed in the community, indicating the presence of challenges and struggles associated with the condition. These findings emphasize the importance of addressing the wellbeing of individuals living with bipolar disorder and highlight the need for appropriate treatment and support. The research framework employed in this study can also be applied to gain insights into other health conditions through social media analysis.

In summary, the primary objective of the present study is to enhance the understanding of how individuals with BD communicate on social media platforms. While not providing definitive medical insights, our research offers opportunities for further exploration. We intend to investigate unmet information needs and aspects requiring professional attention in future research, guided by the input of clinicians and individuals with BD, ensuring practical relevance.

## Data availability statement

The original contributions presented in the study are included in the article/supplementary material, further inquiries can be directed to the corresponding author.

## Ethics statement

The data used in this study was collected from Reddit, a social media platform, in accordance with the platform's terms of service and community guidelines. The data collection process strictly adhered to the ethical standards outlined by Reddit, ensuring respect for user privacy, anonymity, and the responsible use of the platform's content. Consequently, we confirm that the collection, analysis, and reuse of social media data were conducted in strict accordance with Reddit's policies and terms of use, as well as all relevant institutional regulations.

## Author contributions

TT conceived and co-designed the analysis, collected the data, and co-wrote the paper. QX co-designed the analysis and co-wrote the paper. SL co-wrote the paper. All authors contributed to the article and approved the submitted version.
